# A Pragmatic Transfer Learning Approach for Oxygen Vacancy Formation Energies in Oxidic Ceramics

**DOI:** 10.3390/ma15082879

**Published:** 2022-04-14

**Authors:** Xiaoyan Yin, Robert Spatschek, Norbert H. Menzler, Claas Hüter

**Affiliations:** 1 Institute of Energy and Climate Research IEK-2, Forschungszentrum Jülich GmbH, 52425 Jülich, Germany; xiaoyan.yin11@gmail.com (X.Y.); claashueter@posteo.de (C.H.); 2 Institute of Energy and Climate Research IEK-1, Forschungszentrum Jülich GmbH, 52425 Jülich, Germany; n.h.menzler@fz-juelich.de

**Keywords:** solid oxide cells, oxygen vacancy formation energy, transfer learning, artificial neural networks, Bayesian analysis

## Abstract

Lower oxygen vacancy formation energy is one of the requirements for air electrode materials in solid oxide cells applications. We introduce a transfer learning approach for oxygen vacancy formation energy prediction for some ABO${}_{3}$ perovskites from a two-species-doped system to four-species-doped system. For that, an artificial neural network is used. Considering a two-species-doping training data set, predictive models are trained for the determination of the oxygen vacancy formation energy. To predict the oxygen vacancy formation energy of four-species-doped perovskites, a formally similar feature space is defined. The transferability of predictive models between physically similar but distinct data sets, i.e., training and testing data sets, is validated by further statistical analysis on residual distributions. The proposed approach is a valuable supporting tool for the search for novel energy materials.

## 1. Introduction

Reducing CO${}_{2}$ emissions has become a top priority for economies all over the world to fulfill the obligations for environmental protection. Therefore, the development of new, cost-efficient energy conversion technologies becomes significantly important. One such technology is the solid oxide cell (SOC), which either transforms chemical to electrical energy with high efficiency (SOFC: solid oxide fuel cell) or uses renewable energy to produce hydrogen or hydrogen-based synthetic fuels (SOEC: solid oxide electrolysis cell). Additionally, the SOC can also be operated within one device in both modes alternately (rSOC: reversible solid oxide cell) [1,2]. The most important energy carriers include hydrogen, natural gas, biogas and other renewable fuels, and the resulting reduction of emissions of CO${}_{2}$ is a major argument for the large-scale deployment of this technology. However, degradation effects in both modes are still a relevant R&D topic. Especially in SOFC mode, the degradation of the air electrodes has been identified as a major issue limiting the lifetime and durability of the stacks, posing a crucial challenge to extended application.

(La,Sr)(Co,Fe)O${}_{3-\delta }$ (LSCF) is one of the major air electrode materials for SOC applications [3,4,5,6,7,8,9,10,11]. However, Sr is a very reactive element and contributes to several degradation issues. On the one hand, Sr-containing compounds tend to segregate at the LSCF surface in the form of SrO [12,13,14]. In SOFC operation mode, the segregated Sr becomes a reaction partner for volatile Cr species, which are originating from the high chromium-containing metallic interconnects. During the operation of an SOFC stack, a Cr${}_{2}$O${}_{3}$-containing scale forms on the surface of the ferritic interconnect, which results in the evaporation of Cr species. The segregated SrO reacts with the volatile Cr species, forming Sr-Cr-O secondary phases, and leads to poisoning of the LSCF cathode [15]. The typical Cr poisoning product is SrCrO${}_{4}$, which is found on the top of the electrode surface [16]. Due to local drops of the oxygen partial pressure pO${}_{2}$ in the air electrode, Sr-Cr-O also can form at the LSCF/GDC interface [17]. On the other hand, in both SOFC and SOEC operation modes, Sr may diffuse to the ZrO${}_{2}$-based electrolyte through grain boundaries of the Gd-doped Ceria (GDC) diffusion barrier layer, and it may subsequently react with ZrO${}_{2}$ in the electrolyte, forming SrZrO${}_{3}$ at the GDC/electrolyte interface. The formed SrZrO${}_{3}$ is an ionic insulator, which lowers the electrochemical performance of SOCs [1,18]. Additionally, both thermodynamic calculations and experiments [19] show that in the presence of humidity in the air, volatile Sr species (mainly Sr(OH)${}_{2}$) can form, which are similar in amount to the volatile Cr species. The evaporation of Sr leads then to Sr depletion in the LSCF electrode and subsequently lowers the performance. During the long-term operation in SOFC mode, the volatile Sr(OH)${}_{2}$ may react with ZrO${}_{2}$-based electrolytes and form ion insulating SrZrO${}_{3}$ precipitates. Altogether, the negative impact of Sr-related SOC poisoning effects is therefore substantial.

For the long-term stability of SOC stacks, it is essential to suppress detrimental side reactions as much as possible. Therefore, it is desirable to find new, Sr-free air-electrode materials. The potential materials should be chemically and mechanically compatible with adjacent components, possess good tolerance with respect to impurities in the surrounding atmosphere, and also have a low oxygen vacancy formation energy to guarantee fast oxygen surface exchange and bulk diffusion. Perovskite oxides (ABO${}_{3}$) with cubic symmetry, such as La${}_{1-x}$Sr${}_{x}$MnO${}_{3}$ and La${}_{1-x}$Sr${}_{x}$Co${}_{1-y}$Fe${}_{y}$O${}_{3}$, have shown high potential as air electrode materials in SOC applications [20]. In general, the vast amount of candidate materials for potential air electrode materials makes a systematic search for new and superior materials difficult or even impossible, and therefore, guidance by machine learning tools, in particular artificial neural networks (ANNs) for optimising properties of materials is a promising approach.

In this work, we assess the transferability of ANN parameters learned to predict oxygen vacancy formation energies for some ABO${}_{3}$ perovskite systems from two-species-doped to four-species-doped systems. In Section 2, the data sets are introduced, and the methodological background is presented. In Section 3, the feature space for the four-species-doped perovskites is introduced. The transfer learning performance of the sets of models learned for the predictive task is presented, and the statistical analysis required to judge the transferablity of the learned models between the distinct data sets is explained. Both classical hypothesis testing-based and Bayesian sampling approaches are discussed. In Section 4, we summarise and conclude the results for the prediction task, the transfer learning performance and the overall feasibility of this approach.

## 2. Methods and Data

### 2.1. Data Set

**Figure 1:**
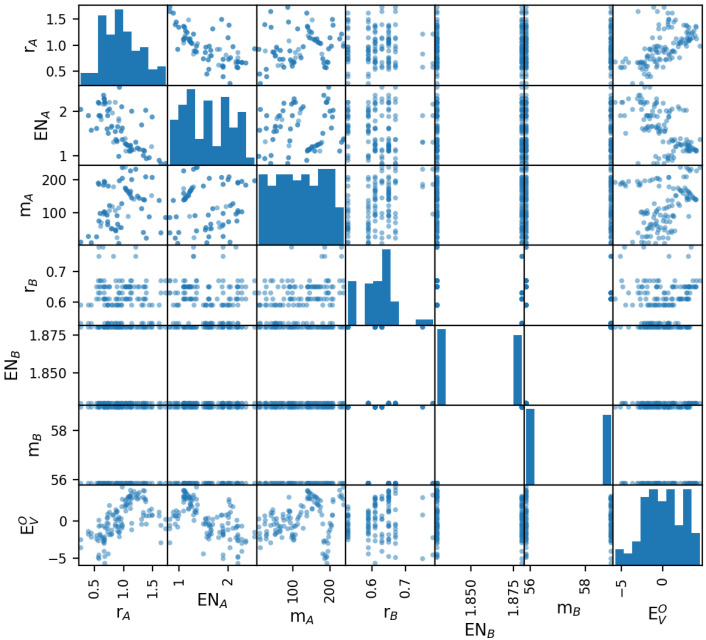
Scatter matrix of the training data set prior to reduction, which allows for an intuitive judgement of pairwise correlations. The units of the features are Ångstrom for the radii, eV/atom for the oxygen vacancy formation energy, and atomic mass units for the mass. The electronegativity is measured using the dimensionless Pauling scale.

The training data are taken from a substantial amount of already published results [21], which contain information on a large variety of ABO${}_{3}$ perovskites. We notice that all the information from [21] was obtained by ab initio calculations. Here, we are specifically interested in ACoO${}_{3}$ and AFeO${}_{3}$ systems, with 121 samples extracted. The features of the data set include the species’ electronegativities $EN_{A}$, $EN_{B}$ (dimensionless Pauling scale), the ionic radii $r_{A}$, $r_{B}$ (in Ångstrom), their masses $m_{A}$, $m_{B}$ (atomic mass units) and others. We stick to them as they represent the selection of features we worked with. Apparently, also the oxygen vacancy formation energy $E_{V}^{O}$, which is the target feature of the prediction task, is provided. The scatter matrixplot of the data set shown in Figure 1 gives a first intuitive impression of the pairwise correlations. The usability of these characteristic features of chemical species for machine-learning based prediction of formation energies was already demonstrated in [22].

The test data set includes 15 A-site doped samples A${}_{1_{x}}$A${}_{2_{1-x}}$(Co, Fe)O${}_{3}$, which are obtained from the literature as listed in Table 1. While the feature space of the training data set directly represents the input features, the feature space of the testing data set represents combinations of physically distinct quantities as joint input features. The layout of the testing data set feature space is detailed in Section 3.1.

### 2.2. Artificial Neural Networks (ANNs) and Transfer Learning

Artificial Neural Networks (ANNs) [27] belong to the class of biologically inspired computational models. Due to their versatility, numerous specialisations have been developed, which all have in common that they are applied to solve complex non-linear problems. Exemplary use cases for materials science have been documented, e.g., in [28,29,30,31,32].

The flexibility of ANNs comes at a price—they belong to the group of rather data-hungry models. Due to the number of parameters that need to be optimized during the calculations, including the selection of the network topology, the required data set size for a generalizable model of certain accuracy is comparably large in relation to less complicated models. This is reflected in the learning curves when benchmarking such models, see, e.g., [33].

The main idea of transfer learning is the re-use of knowledge acquired from one task for a related one [34]. The motivation behind transfer learning includes the size limitations of data sets and also the evolution of the underlying distributions in data sets over time, which poses substantial challenges to many supervised machine learning paradigms. In the context of material science, many data sets used for machine-learning-based investigations of phenomena which are expensive to be addressed experimentally exhibit a small amount of data points. In this sense, this limiting factor for the application of many machine learning approaches is also present in the data set of our interest on doped A${}_{1_{1-x}}$A${}_{2_{x}}$(Co,Fe)O${}_{3}$ perovskites. Another challenging aspect of the SOC data set used in this work is the change of features which we undertake when performing the prediction step, as will be pointed out later. This aspect is typically also addressed by means of transfer learning, e.g., in cross-domain sentiment classification in product reviews [35].

The application of transfer learning requires decisions on the determining aspects of the transfer approach. In the context of our investigations, the aim is the transfer of an ANN model adjusted to a training domain to a target domain for predictions of more general chemical compositions. It is assumed that there is a strong correlation between the two domains in terms of similar distribution functions, which will be also addressed from a statistical point of view in Section 3.3. Therefore, the approach of our choice is categorised as transductive transfer learning, i.e., the source and target domain of the predictive task are different, but related, and the predictive tasks in the source and the target domain are identical.

The criterion for the distinction of different but related domains is the probability distribution function for a given feature set. While the number of features in our training and testing data sets are equal, the physical information associated to the features differs. Therefore, we do not consider identical but rather formally similar domains. As the distribution functions in the training domain and the target domain refer to the same number of phenomenologically similar features, we expect them to be comparable in terms of classical tests for equality of underlying distributions. The relevant quantities for this purpose are the residual distribution of the predictions on the testing data set, which are obtained by applying the transferred ANN model from the training domain and the residual distribution of the training error.

For the regression tasks to which the ANNs are trained, a reasonable model approximation is expected if three main criteria are met. First, the residual distribution has zero mean, i.e., there is no remaining shift or offset between predictions and real values. Second, the residual magnitude is much smaller than the magnitude of the predicted values, and third, the residual distribution function lacks any systematic trend, i.e., is homoscedastic. The validation of all these criteria mentioned here will be described in detail in Section 3.3.

### 2.3. Bayesian Analysis

We use a Bayesian analysis to deepen the understanding of possible discrepancies in the distribution of the residual of the training and the testing data set. Generally speaking, Bayesian analysis allows to estimate from which underlying distribution a present data set could most likely be generated. For this purpose, the parameters that describe the possible distributions are considered as random variables themselves. Thus, the values that these parameters take are randomly varied when generating a large set of corresponding random values. The parameters that characterise the distribution with the highest agreement between randomly generated data and real data are used as approximation of the assumed underlying distribution of the process that generated the real data. Formally, determining the set of characterising parameters that maximises the agreement between generated and real data is based on Bayes’ theorem for distributions:

(1) $$f\left(\theta |\mathrm{data}\right)=\frac{f(\mathrm{data}|\theta )f(\theta )}{f(\mathrm{data})},$$

with f(θ|data) denoting the posterior distribution for the parameter set θ, f(data|θ) denoting the sampling density of the data, which is proportional to the likelihood, f(θ) is the prior distribution for θ and f(data) is the marginal probability of the data.

Practically, employing Bayes’ theorem means that we can simulate f(data|θ) via probabilistic descriptions. For that, we inform the model with a prior f(θ) that reflects our initial understanding of the distribution of the parameter set θ independent of the data set we are operating with. Thus, we can determine the quantity of our interest, namely the posterior distribution f(θ|data). The normalising factor f(data)=∫dθf(data|θ)f(θ) is a constant, and the basic proportionality can be read as Posterior ∼ Likelihood × Prior.

This approach has the substantial benefit that not only point measures such as median or average can be compared, but the entire set of distributional characteristics can be considered. In the context of the investigations presented here, the relevant quantity is the residual distribution in training and testing data sets. As will be pointed out later, a t-distribution will be a suitable basis for the description of the residual in the training data set, as the test data set is small. Therefore, we will also choose a t-distribution when we perform Bayesian analysis on the testing data set and consider the agreement between the distributions on training and testing data set.

The technical basis of the Bayesian analysis presented in Section 3.3.2 is the package pymc3 [36], which is a comprising Python library for Monte Carlo-based sampling schemes. It also wraps the majority of the algorithmic and numerical complexity of implementing efficient solvers that mimic naive Bayesian sampling. Typically (and in our case as well), the applied solvers belong to the class of Markov Chain Monte Carlo (MCMC) algorithms.

## 3. Results and Discussion

### 3.1. Feature Space Reduction and Transfer Feature Space

As explained in Section 2.2, the appropriate choice of the feature space is of decisive importance when learned model parameters shall be transferred for a new predictive task. Based on the available input features, we have evaluated the performance of six input features and the six combinations of five input features when one feature is removed. The difference of the ${\mathrm{MSE}}_{121}$, i.e., the mean squared error (MSE) obtained during the training on the 121 data points, for the six-input feature scenario and the distinct five-input feature options is labelled as $\delta ={\mathrm{MSE}}_{121}-{\mathrm{MSE}}_{121}^{0}$, with ${\mathrm{MSE}}_{121}^{0}$ being the MSE for the six-input feature space. Therefore, the more positive the value of delta is, the more important the removed feature is.

While the reduction of the feature space is typically addressed via unsupervised or semi-supervised machine learning methods such as principle component analysis or manifold reconstruction, our rather manual approach provides a better intuitive understanding of the resulting performance differences. Since the transfer from the two-species-doped perovskites to the four-species-doped perovskites requires a redefinition of the input features, we stay with the manual approach. The input features of these 15 samples are calculated by weighted summation. The weights for the different features are chosen from a set of ad hoc estimates of suitable candidate weights. For example, for La${}_{0.75}$Sr${}_{0.25}$FeO${}_{3}$, suitable weights for estimating the ionic radius $r_{A}$ are

(2) $$r_{A}=0.75r_{La}+0.25r_{Sr}.$$

For each of these six possibly removed features and the full feature set, the topology of the ANN is optimised, using the Tensorflow library [37]. Some qualitative reasoning for the minimal set of neural layers for a given set of activation functions might be inspired by the phenomenological perspective on the problem. However, a precise, quantitative prediction of the optimal ANN topology based on arguments from the application domain is rarely possible. Taking into account that we aim at the regression of the non-linear, yet only mildly complex (considering the dimensionality of the feature space) functional dependence of the oxygen vacancy formation energy on the features introduced in Section 2.1, we tackle this problem as follows: The input layer of the ANN has either five or six nodes, respectively. In both cases, we add nodes and layers sequentially as long as our performance metric of the prediction increases. Therefore, the initial topology on top of the input layer is a hidden layer with two neurons and an output layer with a single neuron. The number of edges ranges from 12 for a 5×2×1 ANN to 66 for a 5×5×5×1 + 11 bias nodes ANN when using five input features. When six input features are used, the number of edges ranges from 14 for an 6×2×1 ANN up to 71 for an 6×5×5×1 + 11 bias nodes ANN.

Taking into account the limited size of the data set and the generalisability of the learned models, the maximum number of neurons is reached quite fast. The size of 121 data points on the training data set leads to a substantial penalty in the bias-corrected performance metrics that we use for the largest topology with altogether 71 edges.

To determine the optimal ANN topology for each of the input feature combinations, a 10-fold cross-validation based on the MSE is used, with the results being listed in Table 2. From these evaluations, the most suitable feature space for the task could be identified. Option 4 has the smallest values for MSE${}_{121}$ (0.675), MSE${}_{15}$ (0.064) and MAPE${}_{15}$ (0.086). Here, MSE${}_{15}$ and MAPE${}_{15}$ are mean squared and mean absolute percentage error on the 15 data points in the test data set. It also has the highest benefit from reducing the feature space, δ=−0.144. This is the only model that provides an MAPE below 10% for the transferred prediction of four-species-doped perovskites from two-species-doped perovskites. For a better impression on the accuracy of the network forecast, we added selected predicted oxygen vacancy formation energy for Ba${}_{x}$Sr${}_{1-x}$Fe${}_{y}$Co${}_{1-y}$O${}_{3}$ (BSCF) to Table 1. We see that the energies and trends are predicted rather well despite the small size of the training data set, which contains only four compositions with this chemistry.

The application of the transfer model to generate a prospective data set is discussed in the next paragraph. From a chemical perspective, we find that the electronegativity, ionic radius and atomic mass of the A-site element are more important features than the corresponding B-site properties. In fact, the electronegativity of the A-site element is the most important feature variable, while the ionic radius of the B-site element is the least important feature variable. The importance of the electronegativity and mass of the B-site element are roughly comparable. Altogether, the feature importance in descending order is: electronegativity of the A-site element, ionic radius of the A-site element, atomic mass of the A-site element, electronegativity of the B-site element, mass of the B-site element and finally the ionic radius of the B-site element. For some parameters, such as the ionic radii or the electronegativity, an influence on the oxygen vacancy formation energy is plausible, as these parameters directly influence the lattice stability and the interatomic bonding. This is less obvious for the masses of the elements, which according to the obtained results do have an influence on $E_{V}^{O}$, although the effect is not as pronounced as in particular that of $EN_{A}$ and $r_{A}$.

### 3.2. Prospective Studies

**Figure 2:**
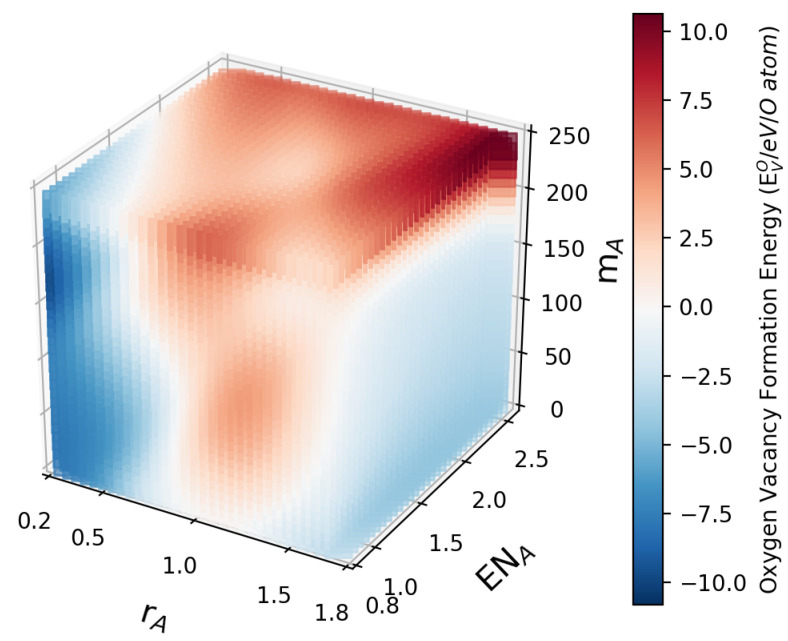
The predicted variation of $E_{V}^{O}$ for A${}_{1_{x}}$A${}_{2_{1-x}}$Co${}_{0.5}$Fe${}_{0.5}$O${}_{3}$ dopings with varying $r_{A}$, $EN_{A}$ and $m_{A}$ for fixed $EN_{B}$ and $r_{B}$. The high and low oxygen vacancy formation energy regions show some clustering, although a simple trend cannot be observed.

As the model transfer from two-species doping to four-species doping has been tested with an acceptable accuracy for the prediction of the oxygen vacancy formation energies, we apply the model next to provide prospective studies for a larger variety of dopings. The first application focuses on A${}_{1_{x}}$A${}_{2_{1-x}}$Co${}_{0.5}$Fe${}_{0.5}$O${}_{3}$ dopings. In the training data set, A${}_{1}$ and A${}_{2}$ are two arbitrary elements from 68 options for the A site. The electronegativity for B (dimensionless Pauling scale) and the mass (in atomic mass units) for B are fixed to 1.855 and 57.3891, respectively, which corresponds to the equal mixing of Fe and Co on this site. Using the trained model, we vary independently the (mean) atomic radius, the electronegativity and the mass of the A site element. Here, we note that only the averaged quantities of the A site elements are needed, which are calculated in analogy to Equation (2) using the values for A${}_{1}$ and A${}_{2}$ and the composition variable *x*, e.g., $m_{A}=xm_{A_{1}}+\left(1-x\right)m_{A_{2}}$. The varied parameters are discretized over 30 steps, with $r_{A}$ being in the range of 0.27 to 1.73 Ångstrom, $EN_{A}$ in the interval [0.82,2.55] of the dimensionless Pauling scale, and $m_{A}$ in the range of 6.941 to 238.0289 atomic mass units, which corresponds to the parameter regimes of typical elements for the A site. The resulting prediction for the oxygen vacancy formation energy $E_{V}^{O}$ of A${}_{1_{x}}$A${}_{2_{1-x}}$Co${}_{0.5}$Fe${}_{0.5}$O${}_{3}$ for given $r_{A}$, $EN_{A}$ and $m_{A}$ is plotted in the heat map shown in Figure 2.

**Figure 3:**
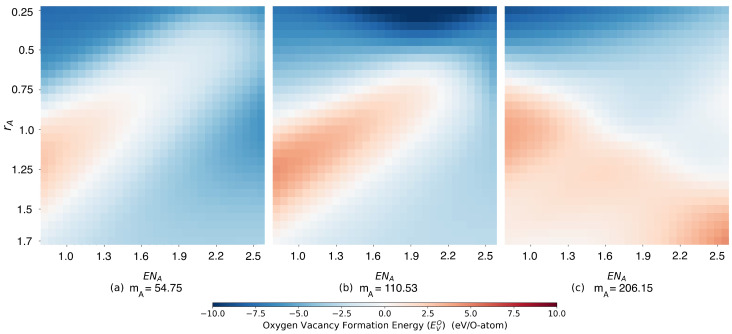
Cuts through the 3D color map in Figure 2 for fixed average A site mass $m_{A}$ for a perovskite of type A${}_{1_{x}}$A${}_{2_{1-x}}$Co${}_{0.5}$Fe${}_{0.5}$O${}_{3}$. Left panel $m_{A}=54.75$, center panel $m_{A}=110.53$ and right panel $m_{A}=206.15$ atomic units.

Obviously, there is some clustering of low and high oxygen vacancy formation energies, but the dependencies are rather nontrivial. For example, low values of $E_{V}^{O}$ can be found in regions of low and high A site radii as well as for low atomic masses $m_{A}$, as can also been seen in two-dimensional slices of the heat map for fixed average A site mass $m_{A}$ in Figure 3.

We note that negative values of the oxygen vacancy formation energies are contained in the ab initio training data and shall typically indicate the instability of the given structure. This aspect will also be discussed below. In the following, we focus on the optimisation of $E_{V}^{O}$, irrespective of the sign.

**Figure 4:**
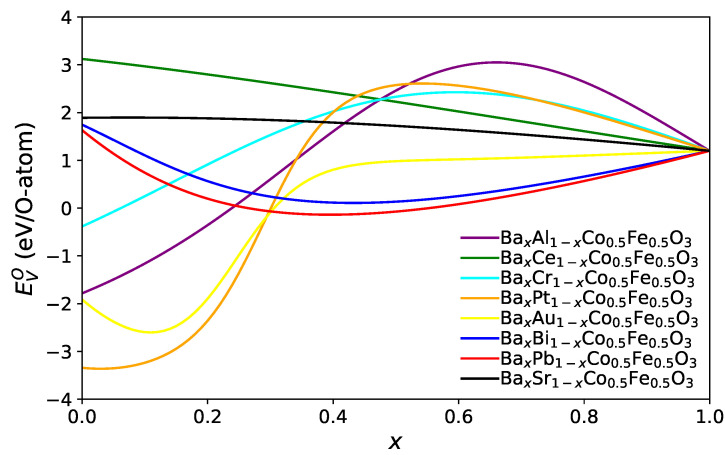
Oxygen vacancy formation energy $E_{V}^{O}$ as predicted from the ANN for potential material combinations A${}_{1_{x}}$A${}_{2_{1-x}}$Co${}_{0.5}$Fe${}_{0.5}$O${}_{3}$.

To link the systematic scan of A site parameters to chemical element, we use the model to predict the oxygen vacancy formation energy for a composition A${}_{1_{x}}$A${}_{2_{1-x}}$Co${}_{0.5}$Fe${}_{0.5}$O${}_{3}$ for all possible pairs A${}_{1}$ and A${}_{2}$. The dependence on the concentration *x* is shown for selected cases of Ba containing compounds in Figure 4.

**Figure 5:**
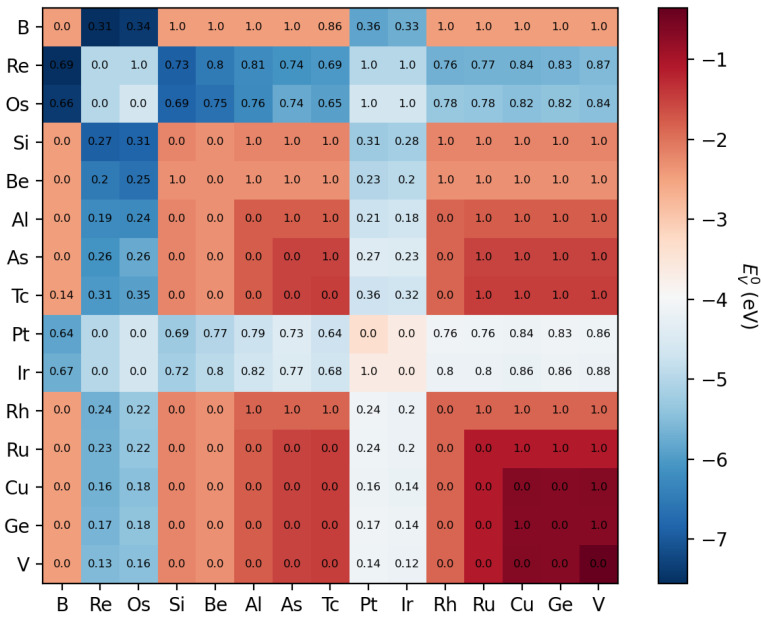
Heat map of the colour-coded oxygen vacancy formation energy $E_{V}^{O}$ for perovskites with the composition A${}_{1_{x}}$A${}_{2_{1-x}}$Co${}_{0.5}$Fe${}_{0.5}$O${}_{3}$. Here, element combinations for A${}_{1}$ and A${}_{2}$ are used, which lead to particularly low values of $E_{V}^{O}$. The numbers inside the element combination field correspond to the composition *x*, which minimises $E_{V}^{O}$.

For many combinations, the minimum oxygen vacancy formation energy $E_{V}^{O}$ is found at the limiting values either x=0 or x=1 (this is the case, e.g., for combinations of alkaline and alkaline earth elements), but sometimes, the functional form is very non-monotonic and exhibits one or several extrema. We have determined for each combination the minimum value of $E_{V}^{O}$ and visualised it in Figure 5 for combinations of selected elements, which lead to particularly low oxygen vacancy formation energies.

Obviously, this matrix is symmetric concerning $E_{V}^{O}$ (the colour coding), as by construction, this predicted value only depends on the weighted average of the ionic radius, electronegativity and mass of the A site elements. Diagonal elements correspond to pure A site occupation, and off-diagonal elements have the antisymmetry property x→1−x when A${}_{1}$ and A${}_{2}$ are exchanged. A striking observation is that for many A site pairs, the energy $E_{V}^{O}$ is minimised indeed for pure elements (x=0 or x=1), and only for specific elements such as Re, Os, Pt, and Ir, a substantial decrease of $E_{V}^{O}$ can be obtained compared to pure elements by forming binaries on the A site, i.e., 0<x<1.

**Figure 6:**
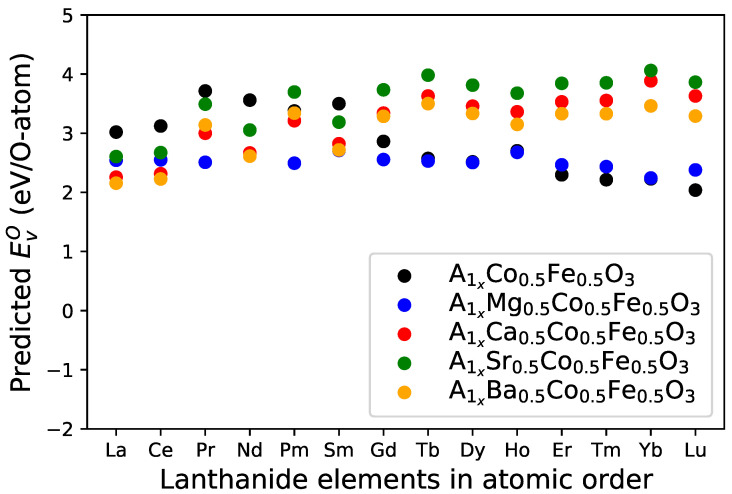
Comparison between the predicted oxygen vacancy formation energy $E_{V}^{O}$ of A${}_{1}$Co${}_{0.5}$Fe${}_{0.5}$O${}_{3}$ perovskites and A site-doped compounds A${}_{1_{0.5}}$A${}_{2_{0.5}}$Co${}_{0.5}$Fe${}_{0.5}$O${}_{3}$ system (A${}_{1}$ = lanthanide element and A${}_{2}$ = alkaline earth metal: Mg, Ca, Sr or Ba).

The second group of prospective calculations focuses on A${}_{1_{0.5}}$A${}_{2_{0.5}}$Co${}_{0.5}$Fe${}_{0.5}$O${}_{3}$ systems, with A${}_{1}$ being a lanthanide element and A${}_{2}$ representing a candidate element from the alkaline earth metals, specifically Mg, Ca, Sr and Ba. The resulting predictions are shown in Figure 6.

Generally, when A${}_{1}$ belongs to the group of the first six lanthanide elements, counted from La to Sm, doping with 50% Mg or Ca or Sr or Ba at the A site leads to a reduction of $E_{V}^{O}$. Here, we should mention that Pm${}_{0.5}$Sr${}_{0.5}$Co${}_{0.5}$Fe${}_{0.5}$O${}_{3}$ is an exception. If A${}_{1}$ corresponds to one of the last eight lanthanide elements, counting from Gd to Lu, doping with 50% Ca or Sr or Ba at the A site increases the oxygen vacancy formation energy $E_{V}^{O}$.

**Figure 7:**
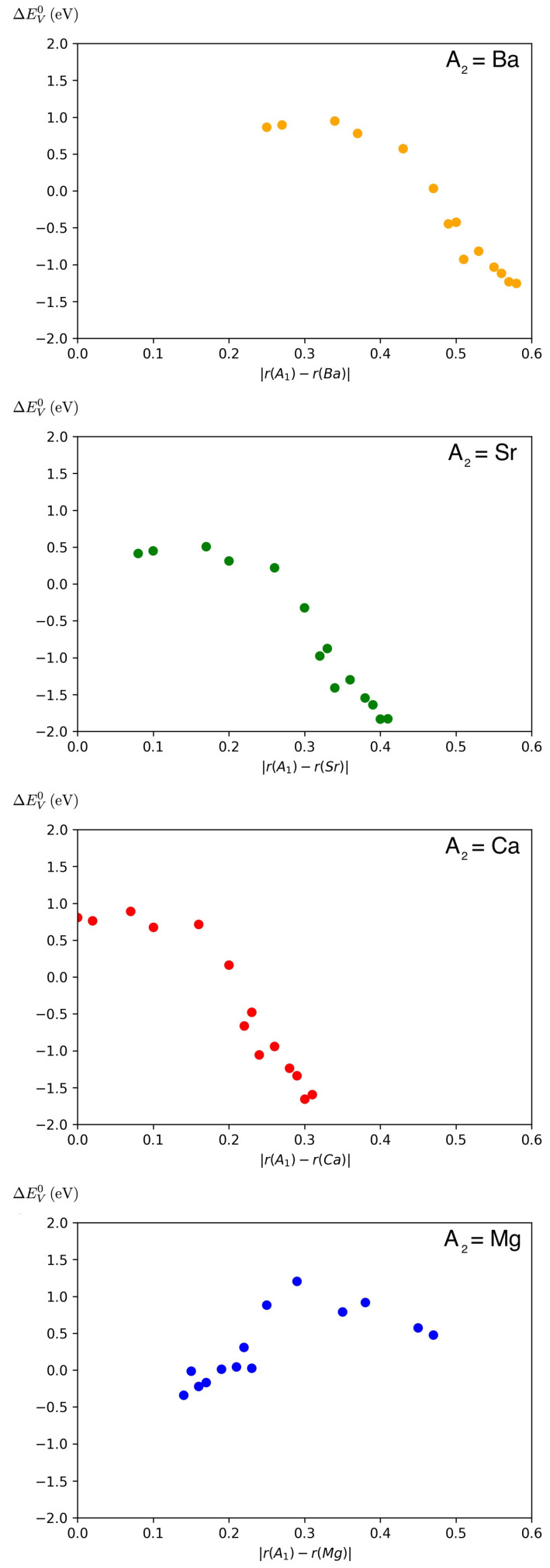
The influence of the ionic radius difference between the host element (A${}_{1}$ = lanthanide element) and the dopant element (A${}_{2}$ = Mg, Ca, Sr and Ba) at the A site on the oxygen vacancy formation energy $E_{V}^{O}$.

Apart from the influence of the doping for varying chemical composition, also the dependence of $E_{V}^{O}$ on the ionic radius difference between host element A${}_{1}$ and doping element A${}_{2}$ has been investigated. As shown in Figure 7, with an exception for Mg as a dopant, there is a robust trend for the dependence of the oxygen vacancy formation energy difference on the ionic radius difference. The oxygen vacancy formation energy difference is calculated as:

(3) $$\Delta E_{V}^{O}=E_{V}^{O}\left(A_{1}{\mathrm{Co}}_{0.5}{\mathrm{Fe}}_{0.5}O_{3}\right)-E_{V}^{O}\left(A_{1_{0.5}}A_{2_{0.5}}{\mathrm{Co}}_{0.5}{\mathrm{Fe}}_{0.5}O_{3}\right)$$

For Ca, Sr and Ba as dopants, the difference in the vacancy formation energy shifts from the positive to the negative regime with increasing difference of the ionic radii of host and dopant. For Mg, no clear trend is visible. Altogether, these observations support the role of the ionic radius as a relevant feature variable for the oxygen vacancy formation energy.

Altogether, preferable air electrode materials for SOCs exhibit low values of $E_{V}^{O}$, as they correspond to a higher concentration of oxygen vacancies. This promotes rapid oxygen adsorption and dissociation at the electrode.

However, to be a potential air electrode material, there are other conditions that should be also considered, such as the stability of the perovskite structure, a considerable electronic conductivity, and the chemical and mechanical compatibilities with other components in SOCs. The stability of the structure is also related to the sign of $E_{V}^{O}$, and it is obvious that a comprehensive investigation requires also the consideration of such criteria, which is beyond the scope of the present work.

### 3.3. Statistical Analysis: Validity of the Applied Transfer Learning Approach

As discussed in Section 2.2 on artificial neural networks, performing regression via ANNs is in general a non-linear regression. Therefore, the standard approach to estimate the quality of a fit and significance of a regression in terms of $R^{2}$ and a *p*-value is not generally applicable. Furthermore, we use very asymmetrically sized training and test data sets while assuming we may transfer the model learned on the training data to apply it to the test data based on the formal similarity of the feature spaces for both data sets. The most crucial point that must be addressed concerns the transferability of the trained model to the testing data set. In the ABO${}_{3}$ training data set with binary doping, the B site is either Fe or Co, and the determined characteristic is the set $\left[r_{A},EN_{A},m_{A},EN_{B},m_{B}\right]$. In the testing A${}_{1}$A${}_{2}$B${}_{1}$B${}_{2}$O${}_{3}$ data set, the same characteristic is used, but the radius, electronegativities and masses are obtained as linear superposition in accordance with the stochiometry of the sample. While the format of the features remains unchanged, it is not clear if the choice of linear superposition for the complex doped configurations in the test sample is suitable for the prediction of the oxygen vacancy formation energy. Usually, in applications of transfer learning, the transferred model parameters are used as initialisation of another training of the transferred model, or additional layers are added to adapt the learned model to the new data set. However, due to the limited size of our testing data set, we aim at a direct transfer of the model, which was trained on the 121 ABO${}_{3}$ data points to the test data set, which consists of 15 A${}_{1}$A${}_{2}$B${}_{1}$B${}_{2}$O${}_{3}$ data points. Consequently, it is required to investigate the feasibility of this approach from a statistical perspective. In addition to the performance of the model on the individual data sets, we therefore also evaluate the discrepancy of the residual distributions in both data sets with respect to the trained model. The evaluation is performed both from a classical null hypothesis testing (NHT) and a Bayesian perspective.

#### 3.3.1. Classical Null Hypothesis Testing

The error in both data sets needs to be normally distributed with zero mean and homoscedastic. Satisfaction of these two criteria tells us—independent of the accuracy of the learned models—how reasonable it is to approach the prediction of the oxygen vacancy formation energies via a machine-learned model. In addition to visual inspection, we conduct the Shapiro–Wilk (SW) [38] test for normalcy of the residual distribution, a Breush–Pagan (BP) test for heteroscedasticity and the Kolmogorov–Smirnov test for the difference between the two univariate distributions. We briefly recall the idea and evaluation of these three tests before we present the results.

The Shapiro–Wilk test is a test for the null hypothesis that a data set is normally distributed. The sample should not include more than 5000 data points, and the Shapiro–Wilk test is reported to provide a higher power than other normalcy tests based on analysis of variance (ANOVA) in the regime of small data sets with up to 50 data points [39]. Therefore, it is the test of choice, as we want to use the same test with the same implementation (scikit.stats package) on both data sets for easy comparability. The Shapiro–Wilk test statistic W measures the correlation between the transformed and standardised empirical distribution and the normal distribution and thus has an upper limit of unity. The *p* value of the W statistic has to be larger than the desired alpha level of the test, i.e., the probability of false rejection of the null hypothesis.

Concerning the homoscedasticity assumption, the Breush–Pagan test detects linear deviations from a homogeneous variance distribution. The test statistic LM is defined as $R^{2}$ scaled by the sample size *n*, $LM=nR^{2}$. If it has a *p* value below 0.05, the null assumption of homoscedasticity is rejected. Due to the smallness of the test data set, we prefer it instead of the White test, which is more sensitive as it covers more types of heteroscedasticity but requires a large sample size as it is an asymptotic test.

The third test we apply is the Kolmogorov–Smirnov (KS) two-sample test. It considers the difference in the empirical distribution functions (i.e., probability that the empirical sample from the assumed distribution takes values up to x) $F_{1,n}\left(x\right),F_{2,m}\left(x\right)$, with sample sizes *n* and *m* for training and test data. The test statistic is:

(4) $$D_{n,m}=\sup_{x}\left|F_{1,n}\left(x\right)-F_{2,m}\left(x\right)\right|.$$

The null hypothesis is the equality of the underlying distribution functions, and for a given alpha level, it is rejected for:

(5) $$D>\sqrt{-\frac{1}{2}\ln\frac{\alpha }{2}}\sqrt{\frac{n+m}{nm}}.$$

We note that the Kolmogorov–Smirnov test is robust to small sample sizes and is non-parametric; i.e., while the small data set with 15 data points only allows for a check of normality with less power, the Kolmogorov–Smirnov test would not suffer from deviations from normality. However, as pointed out in [40], the sample sizes for the two-sample KS test should be chosen equally if possible, as increasing asymmetry of the sample sizes leads to higher probability of not rejecting a false null. Therefore, we restrict the training data set to the subset defined by all values which are in the range of the predicted values of the test data set. The resulting restricted training data set contains 38 data points. Consequently, with a confidence level of α=0.01, the critical value of the KS test statistic, D, is $D_{\mathrm{crit}}\left(\alpha =0.01\right)=0.497$. Using the scipy implementation of the two-sample KS test, the value for the KS statistic is D=0.202 with a *p*-value of $p_{D}=0.723$. The KS test does not reject the hypothesis of equal distribution functions as $D<D_{\mathrm{crit}}$, and the *p*-value above the anticipated significance level indicates equality.

We summarize the null hypothesis test-based inspection of the statistical aspects of the training and test data set in Table 3.

The model trained on the training data set satisfies the normalcy of the residual distribution on both data sets as indicated by the Shapiro–Wilk test results. The Breush–Pagan Test tests the systematic linear deviation from a normal distribution of the residuals. It shows a *p* value of 3.83% for the training data set and 2.29% for the test data set, indicating that homoscedasticity is not fulfilled. However, while the scaled $R^{2}$ indicated by the value of the Lagrange parameter LM is about ≈0.08 for the training data set, it is of order 1 for the test data set. For a standard confidence level of α=0.05, the assumption of homoscedasticity thus would be rejected, although we see a small value for the squared coefficient of determination $R^{2}$ for the training data set.

The Kolmogorov–Smirnov two-sample test shows no rejection of the hypothesis of equal underlying distribution of training and test data. While we reduced the difference in sample sizes to take into account the weakness of the KS two-sample test with respect to sample size differences, this is a qualitative approach that would require extensive simulation efforts to be developed as a quantitative scheme. Such simulation efforts are beyond the scope of this article. We note that the KS test statistics are determined for an alpha level of 0.01 to provide a higher requirement on the probability of a false rejection here.

Whereas Breush–Pagan and Kolmogorov–Smirnov testing support the transfer learning approach, the Breush–Pagan test indicates heteroscedasticity especially in the test data set. Therefore, to further investigate the similarity of predictions for training and test data set, the next paragraph provides the results from a Bayesian analysis of the discrepancy of the residual distributions in both data sets. Combining these results and the results from the next section provides a better basis to judge whether the introduced pragmatic transfer learning approach is suitable.

#### 3.3.2. Bayesian Analysis

**Figure 8:**
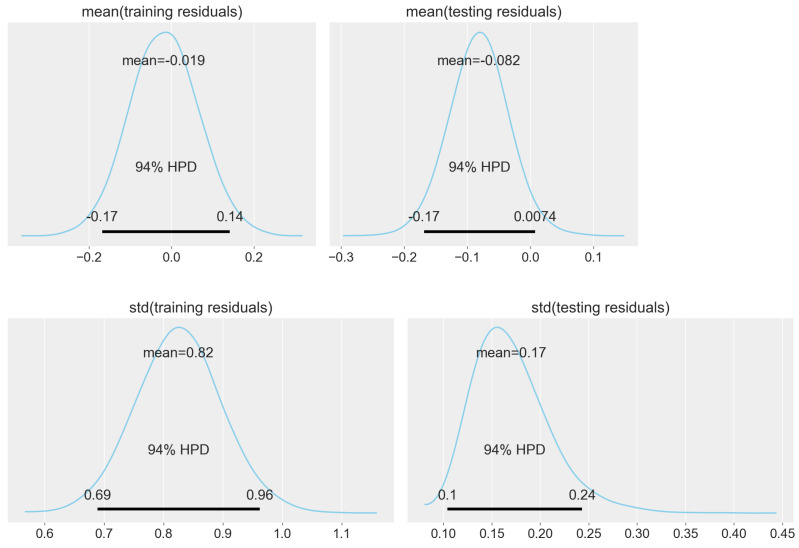
Bayesian assessment of the residuals for the 5 × 5 × 1 + 6 bias ANN layout in the testing and training data set. The top left panel shows the fitting of the normal distribution of the mean of the residual of the training data for an underlying t-distribution. The top right panel shows the mean value normal distribution of the test data residual for an underlying t-distribution. The bottom left right and bottom right panels present the analogous information for the standard deviation. Note that the normality factor of the underlying t-distribution has a mean of ν=13. The shown highest posterior density (HPD) intervals are fixed to 0.94.

The formal basis for the comparison of training and test data set is the residual distribution. If the residual distributions in both data sets are not significantly different, it is assumed that the transfer of the learned parameter values from the training to the testing data set is appropriate. To estimate the distributional discrepancies in both data sets via Bayesian analysis, a sampling distribution has to be chosen. Based on the results presented below in Figure 8, we choose the t-distribution as we see potential finite size effects due to the limited sample size. Therefore, the probabilistic character of the model specification includes not only the mean and standard deviation, as it would in the case of a Gaussian distribution, but also the normality parameter ν. The normality parameter controls the strength of the finite size effects in terms of the tail mass in the t-distribution. When ν→∞, the t-distribution becomes identical to a Gaussian distribution.

The first part of the Bayesian analysis of the transferability of trained parameters from the training to the testing data set includes a probabilistic modelling of both data sets. Therefore, we perform a Bayesian analysis using the library pymc3 for the characteristics of the t-distributions that we use to model the residual distribution. The random variables for the sampling thus include the mean, standard deviation and normality parameter. Generator distributions are a Gaussian for the mean, a half (positive) Gaussian for the standard deviation and an exponential distribution for the normality parameter. To reflect potential finite size effects in the testing data set, we assign one common normality parameter distribution to the generator samples from both the training and testing data set. The results from this first step of the analysis are visualised in Figure 8.

In this figure, the distributions approximating the residuals are also evaluated with respect to the highest posterior density (HPD) regions. It is defined as a credible interval in Bayesian analysis, i.e., the interval into which an unobserved parameter will fall with a certain probability. It is the shortest possible interval on the posterior density for the given probability, in our case 0.94 (for further details, we refer to [41]). As shown in Figure 8, both residual distributions have a comparably small deviation from zero mean, and the HPDs have a large overlap, indicating a high probability that any residual mean obtained from modelling the training sample could also be obtained from modelling the testing sample. In contrast to that, the means and HPDs of the standard deviation modelling of the residuals show a relevant discrepancy, which requires attention in the following step.

**Figure 9:**
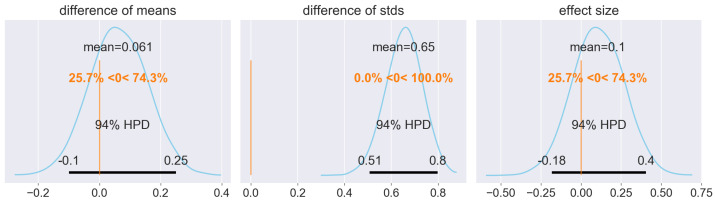
Bayesian assessment of the difference in the underlying generating distributions. In the left panel, the difference of means shows the distributional discrepancy between the residual distribution in the training and testing data set. With a mean shift of only 0.061, both the training and test data sets exhibit only weak deviations from the zero mean assumption for the residual distribution. While the standard deviation shows a more pronounced difference between the simulated t-distributions on the training and test data sets (centre panel), the overall effect size is small with a value of 0.1 (right panel). Note that the choice of the effect size metric corresponds to Cohen’s d, which is a pooled mean shift relative to the standard deviation.

Finally, for the quantitative part of the Bayesian analysis of transferability, the metric for effect size of our choice is Cohen’s d; i.e., it is the ratio of the mean shift to the pooled (symmetrically weighted) standard deviation. Therefore, we consider the distribution of the mean shift between the training and testing data set residual distribution, the distribution of the standard deviation shift between the training and testing data set residual distribution, and the effect size, which is Cohen’s d. With a value of only 0.1, the effect is weak, which suggests that transferability of the trained model from the training to the testing data set is indeed appropriate. The results are shown in Figure 9.

## 4. Conclusions

Finding new materials for energy applications is a complex task with many, often conflicting requirements. Due to the enormous number of elemental combinations, a brute force approach is usually impossible. Therefore, guidance from modelling approaches, and more recently in particular machine learning techniques, can be extremely valuable. One of the challenges for the automatic search for material candidates is typically the low number of data sets, which can be used to train the models. Due to this limitation, an extension towards more general material classes beyond the training data set is usually a major challenge.

The central motivation of our investigation was therefore the transferability of predictive models between physically similar but distinct data sets. Hence, an ANN was trained to predict the oxygen vacancy formation energy $E_{V}^{O}$ of two species doped perovskites ACoO${}_{3}$ and AFeO${}_{3}$, as a basis for transfer learning towards more complex configurations. For that, the ANN parameters learned to predict the $E_{V}^{O}$ from the domain of two-species-doped perovskites (ACoO${}_{3}$ and AFeO${}_{3}$) were directly transferred and used to predict the $E_{V}^{O}$ of four-species-doped perovskites (A${}_{1_{x}}$A${}_{2_{1-x}}$(Co,Fe)O${}_{3}$). We find that the MAPE of the transferred model is less than 10%. Therefore, the transferred model can be used for screening potential perovskite candidates for SOC application regarding the oxygen vacancy formation energy. The transferability was validated by statistical analysis on the residual distributions of both the two-species-doped perovskites (ACoO${}_{3}$ and AFeO${}_{3}$) data set and the four-species-doped perovskites (A${}_{1_{x}}$A${}_{2_{1-x}}$(Co,Fe)O${}_{3}$) data set. For that, classical null hypothesis testing and Bayesian analysis were performed. As examples of applications, at first, the transferred model was used to predict the $E_{V}^{O}$ of A${}_{1_{x}}$A${}_{2_{1-x}}$Co${}_{0.5}$Fe${}_{0.5}$O${}_{3}$ systems, with A${}_{1}$ and A${}_{2}$ being two arbitrary elements from the elements pool. The results show a nontrivial clustering of low and high $E_{V}^{O}$, from which no simple design rules can be derived. Then, the transferred model was used to predict the $E_{V}^{O}$ of A${}_{1_{0.5}}$A${}_{2_{0.5}}$Co${}_{0.5}$Fe${}_{0.5}$O${}_{3}$ systems, with A${}_{1}$ being a lanthanide host element and A${}_{2}$ a dopant element from the alkaline earth metals, specifically Mg, Ca, Sr and Ba. It was found that the ionic radius difference between the host and the dopant elements at the A site has an impact on $E_{V}^{O}$. While the reached level of accuracy may not be sufficient to save final calculations or experiments on promising perovskite candidate systems, it is satisfactory for screening purposes at a small amount of computational expense. Especially for complex materials, where a large difference in computational effort between ab initio simulations and machine learning exists, such a screening approach can offer a striking advantage, as this allows scanning significantly larger configurational spaces. Moreover, the approach may unravel the influence of parameters on the output quantity, for which physical arguments are less obvious. In return, a purely data-based inspection without the use of physical arguments can hardly lead to quantitative descriptions for sparse data sets, which are rather common in materials science. Overall, the developed scheme may therefore be a valuable supporting tool for the search for novel energy materials with optimized properties.

## Figures and Tables

**Table 1:** Oxygen vacancy formation energies and references. The selected predictions in the last column result from the 5×5×1 + 6 bias network (option 4).

Chemical Formula	$E_{V}^{O}$/eV per O-Atom	Reference	$E_{V}^{O}$/eV (Predicted)
La${}_{0.75}$Sr${}_{0.25}$FeO${}_{3}$	3.412	[23]	
La${}_{0.5}$Sr${}_{0.5}$FeO${}_{3}$	3.344	[23]	
La${}_{0.8}$Ca${}_{0.2}$FeO${}_{3}$	3.515	[24]	
Pr${}_{0.8}$Ca${}_{0.2}$FeO${}_{3}$	3.53	[24]	
Ba${}_{0.5}$Sr${}_{0.5}$FeO${}_{3}$	2.22	[25]	2.05
La${}_{0.75}$Sr${}_{0.25}$CoO${}_{3}$	2.30	[23]	
La${}_{0.5}$Sr${}_{0.5}$CoO${}_{3}$	2.19	[23]	
Ba${}_{0.5}$Sr${}_{0.5}$CoO${}_{3}$	1.21	[25]	1.39
La${}_{0.5}$Sr${}_{0.5}$Co${}_{0.5}$Fe${}_{0.5}$O${}_{3}$	2.75	[23]	
La${}_{0.75}$Sr${}_{0.25}$Co${}_{0.5}$Fe${}_{0.5}$O${}_{3}$	2.82	[23]	
La${}_{0.5}$Sr${}_{0.5}$Co${}_{0.8}$Fe${}_{0.2}$O${}_{3}$	2.735	[26]	
Ba${}_{0.5}$Sr${}_{0.5}$Co${}_{0.5}$Fe${}_{0.5}$O${}_{3}$	1.63	[25]	1.72
Ba${}_{0.5}$Sr${}_{0.5}$Co${}_{0.75}$Fe${}_{0.25}$O${}_{3}$	1.35	[25]	1.56
Ba${}_{0.5}$Sr${}_{0.5}$Co${}_{0.25}$Fe${}_{0.75}$O${}_{3}$	1.87	[25]	1.87
SrCo${}_{0.8}$Fe${}_{0.2}$O${}_{3}$	1.59	[26]	1.74

**Table 2:** Selected ANN topologies. MSE${}_{15}$ and MAPE${}_{15}$ are mean squared and mean absolute percentage error on the 15 data points in the test data set.

Feature Variables	Selected ANN Topology	MSE${}_{121}$	MSE${}_{15}$	MAPE${}_{15}$	$\delta ={\mathrm{MSE}}_{121}-{\mathrm{MSE}}_{121}^{0}$
Group 1: $EN_{A}$, $EN_{B}$, $r_{A}$, $r_{B}$, $m_{A}$, $m_{B}$	6×4×1+0 bias	0.819	0.723	0.352	0
Group 2: $EN_{A}$,$EN_{B}$, $r_{A}$, $r_{B}$, $m_{A}$	5×5×1+0 bias	0.753	0.636	0.334	−0.066
Group 3: $EN_{A}$, $r_{A}$, $r_{B}$, $m_{A}$, $m_{B}$	5×5×1+0 bias	0.806	0.859	0.370	−0.013
Group 4: $EN_{A}$, $EN_{B}$, $r_{A}$, $m_{A}$, $m_{B}$	5×5×1+6 bias	0.675	0.064	0.086	−0.144
Group 5: $EN_{A}$, $EN_{B}$, $r_{B}$, $m_{A}$, $m_{B}$	5×5×1+6 bias	1.122	0.123	0.103	0.303
Group 6: $EN_{B}$, $r_{A}$, $r_{B}$, $m_{A}$, $m_{B}$	5×3×3×1+0 bias	1.246	3.152	0.763	0.427
Group 7: $EN_{A}$, $EN_{B}$, $r_{A}$, $r_{B}$, $m_{B}$	5×5×1+6 bias	0.987	0.264	0.226	0.168

**Table 3:** Comparison of relevant test statistics for training and testing data sets. For the Kolmogorov–Smirnov two-sample test, we report the difference of the empirical distribution functions and the *p*-value in both columns, as it describes the difference between these two and thus belongs to both data sets.

Test Statistics	Training Data Set	Test Data Set
Shapiro–Wilk: (W,p)	0.988, 0.389	0.984, 0.989
Breush–Pagan: $\left(LM,p_{\mathrm{LM}}\right)$	10.13, 0.0383	11.35, 0.0229
Kolmogorov–Smirnov: $\left(D,p_{D}\right)$	0.202, 0.723	0.202, 0.723

## Data Availability

Not applicable.
